# Shared mechanisms of neural circuit disruption in tuberous sclerosis across lifespan: Bridging neurodevelopmental and neurodegenerative pathology

**DOI:** 10.3389/fgene.2022.997461

**Published:** 2022-11-25

**Authors:** Karen Litwa

**Affiliations:** Department of Anatomy and Cell Biology, Brody School of Medicine, East Carolina Diabetes and Obesity Institute, East Carolina University, Greenville, NC, United States

**Keywords:** synapse, tau, mTORC, tuberous sclerosis, GABA, neurodevelopment, neurodegeneration

## Abstract

Tuberous Sclerosis (TS) is a rare genetic disorder manifesting with multiple benign tumors impacting the function of vital organs. In TS patients, dominant negative mutations in *TSC1* or *TSC2* increase mTORC1 activity. Increased mTORC1 activity drives tumor formation, but also severely impacts central nervous system function, resulting in infantile seizures, intractable epilepsy, and TS-associated neuropsychiatric disorders, including autism, attention deficits, intellectual disability, and mood disorders. More recently, TS has also been linked with frontotemporal dementia. In addition to TS, accumulating evidence implicates increased mTORC1 activity in the pathology of other neurodevelopmental and neurodegenerative disorders. Thus, TS provides a unique disease model to address whether developmental neural circuit abnormalities promote age-related neurodegeneration, while also providing insight into the therapeutic potential of mTORC1 inhibitors for both developing and degenerating neural circuits. In the following review, we explore the ability of both mouse and human brain organoid models to capture TS pathology, elucidate disease mechanisms, and shed light on how neurodevelopmental alterations may later contribute to age-related neurodegeneration.

## Introduction

### Overview of Tuberous Sclerosis

Tuberous Sclerosis (TS) is a rare genetic disorder manifesting with multiple benign tumors impacting the function of vital organs (Curatolo et al., 2015). TS is driven by autosomal dominant negative mutations in either of the Tuberous Sclerosis Complex (TSC) genes, *TSC1* or *TSC2* (Laplante et al., 2012). A significant portion of patients also exhibit germline and/or somatic mosaicism, resulting in heterogenous expression of *TSC1/2* mutations (Verhoef et al., 1999; Giannikou et al., 2019). In most patients, *TSC1/2* mutations impact central nervous system function, resulting in infantile seizures, intractable epilepsy, and TS-associated neuropsychiatric disorders (TAND), including autism, attention deficits, intellectual disability, and mood disorders (Shepherd and Stephenson, 1992; de Vries and Watson, 2008; Chu-Shore et al., 2010; Bolton et al., 2015; de Vries et al., 2015; Kingswood et al., 2017; de Vries et al., 2018; Cervi et al., 2020). Recently, significant phenotypic overlap between TS and frontotemporal dementia (FTD) has also been described; this overlap includes TAND-associated cognitive-behavioral alterations, as well as clinical biomarkers, such as phosphorylated tau (Olney et al., 2017; Liu et al., 2020a; Alquezar et al., 2021; Liu et al., 2022). These neuropsychiatric manifestations significantly affect quality of life for TS patients and their families, accounting for the majority of disease-associated morbidity, mortality, and burden of care in the TS patient population (Curatolo et al., 2015).

Despite this significant burden, the neuropsychiatric component of TS is the most complex and least understood disease aspect. At a molecular level, TSC1 and TSC2 form a complex that negatively regulates mammalian target of rapamycin complex-1 (mTORC1) -mediated growth pathways (Laplante et al., 2012). Thus, disease-associated mutations result in increased mTORC1 activity and growth, leading to tumor formation (Laplante et al., 2012; Lipton and Sahin, 2014). Not surprisingly, therapeutic approaches have largely focused on mTORC1 inhibitors, such as rapamycin and rapamycin-derivatives. mTORC1 inhibitors, such as the rapamycin derivative everolimus, have demonstrated efficacy in preventing disease-associated tumor growth, including the growth of subependymal giant cell astrocytomas in the brain (Franz et al., 2013; Franz et al., 2015; Lechuga et al., 2019). However, attempts at treating neurocognitive impairment in TS have been mixed (Krueger et al., 2013; Tran et al., 2015; French et al., 2016; Krueger et al., 2017; Overwater et al., 2019). For example, everolimus fails to treat intractable epilepsy in at least 50% of patients (French et al., 2016; Overwater et al., 2019), and there is no clinically observed benefit to TAND (Krueger et al., 2017). This failure of mTORC1 inhibitors suggests tumor-independent mechanisms contribute to neurocognitive impairment. Tumor-independent mechanisms in TS pathology are supported by mouse models, in which pathogenic *TSC1* and *TSC2* mutations give rise to neuron-autonomous alterations in synapse development (Feliciano et al., 2013/02; Feliciano et al., 2020; Bassetti et al., 2021; Bateup et al., 2013). Understanding how TS-associated synaptic alterations contribute to neurocognitive impairment and increased risk of dementia within the TS patient population (Liu et al., 2020a) will have implications for the larger autism spectrum, where synaptic alterations are a common pathological feature (Penzes et al., 2011; Phillips and Pozzo-Miller, 2014). Emerging evidence also suggests that patients within autism spectrum disorders are at an increased risk of early onset dementia (Vivanti et al., 2021). Thus, it is necessary to understand how synaptic alterations within developing circuits may later contribute to synapse loss, neurodegeneration and cognitive decline in dementia. This review addresses shared pathological features linking synaptic dysfunction across TS patient lifespan with the hope of elucidating mechanisms that drive age-related cognitive loss within the larger autism patient spectrum.

### Synapse formation in developing neural circuits

Before we address how synapses are altered in TS, we will first discuss how they typically form during development. Synapses are the points of contact between neurons which facilitate electrochemical communication, giving rise to complex cognitive functions, such as learning, memory, and social behavior (Lynch et al., 2007). In humans, synapse formation begins around mid-fetal gestation (Tau and Peterson, 2010). Synapses can either be inhibitory, suppressing action potential formation, or excitatory, promoting action potential formation. Inhibitory GABAergic synapse formation precedes the formation of excitatory glutamatergic synapses (Ben-Ari, 2002; Ben-Ari, 2006). However, inhibitory synapses initially exhibit GABA-induced excitation (Ben-Ari, 2002; Ben-Ari, 2006). This GABA-elicited depolarization may serve neuroprotective roles in the fetal development of neural circuitry since the excitatory transmitter glutamate can be cytotoxic. However, since GABA is derived from glutamate, this initial GABA-induced excitation may allow GABA to promote neurite and synapse formation, while also preventing glutamate-induced neurotoxicity in vulnerable neural circuits of the developing brain (Ben-Ari, 2002; Ben-Ari, 2006). In immature neural circuits, expression of the sodium-potassium-chloride importer, NKCC1, is high, while expression of the potassium chloride exporter, KCC2, is low (Ben-Ari, 2002; Sernagor et al., 2010; Côme et al., 2019). The ratio of NKCC1 to KCC2 affects the reversal potential of GABA receptors, resulting in Cl^−^ efflux and depolarization when the ratio of NKCC1:KCC2 is high (Liu et al., 2020b). However, KCC2 expression beginning at 18–25 post-conception weeks in subplate and cortical plate neurons enables GABA-induced inhibition through chloride influx and hyperpolarization characteristic of mature circuits (Sedmak et al., 2016).

Corresponding with the developmental shift from GABA-induced excitation to inhibition, excitatory synapses containing pre-synaptic vesicular glutamate transporters and post-synaptic glutamate receptors, begin to form. These excitatory synapses initially form along dendrites or on finger-like dendritic projections, known as filopodia-like spine precursors, but after birth, they are predominantly found on specialized dendritic projections, known as spines (Wilson and Newell-Litwa, 2018). In their mature state, these dendritic spines exhibit a polarized mushroom-shaped structure with a bulbous head atop a thin spine neck (Newell-Litwa et al., 2015). This morphology helps to facilitate action potential propagation. The increased surface area of the head region increases the number of glutamate receptors adjacent to the pre-synaptic axon terminal, thus increasing the likelihood of action potential formation. Furthermore, the physical properties of the thin spine neck alter resistance, with length of the spine neck inversely correlating with action potential formation (Araya et al., 2014; Tønnesen and Nägerl, 2016). Unlike inhibitory synapses which form along the dendritic shaft, the unique morphology of the post-synaptic compartment of excitatory synapses allows them to be readily visualized in post-mortem tissue without the use of immunolabeling, which often requires antigen retrieval in human post-mortem samples. Furthermore, in transmission electron microscopy, the electron-dense post-synaptic density of excitatory synapses is also readily visible (Hahn et al., 2009/04). Because of these attributes, the majority of post-mortem human brain studies of neuropsychiatric disorders have focused on changes to excitatory synapses. In humans, excitatory synapse formation lasts until later juvenile stages (∼5–6years). Throughout adolescence, synaptic pruning refines developing neural circuits, leading to relatively stable synapse densities throughout adulthood, except for cases of age-related cognitive decline and neurodegenerative diseases when synapse numbers once more decline (Penzes et al., 2011).

## Synaptic pathology in TS

**FIGURE 1:**
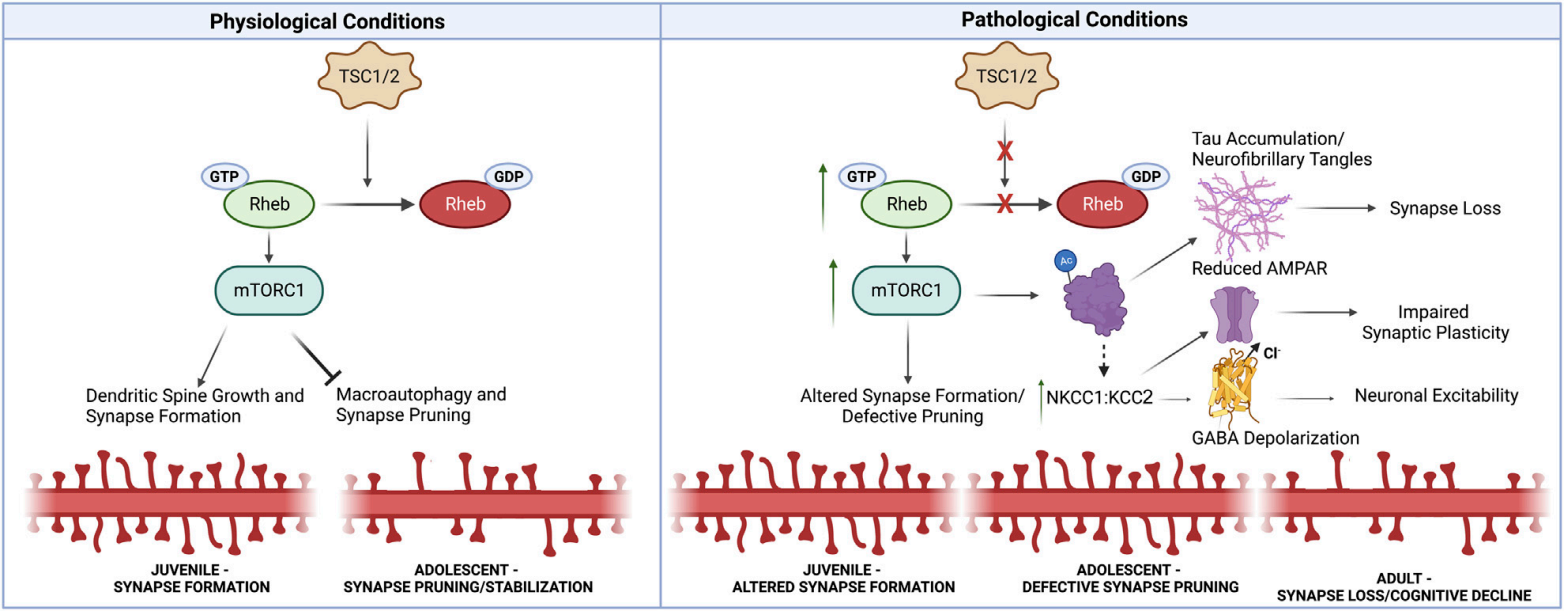
Synaptic Alterations Linking Neurodevelopmental and Neurodegenerative Pathology in TS. Under physiological conditions, TSC1/2 inhibit mTORC1 activity through Rheb-GTPase. mTORC1 promotes synapse formation, but prevents macroautophagy-mediated synaptic pruning later in development. Thus under pathological conditions in TS, mTORC1 activity is increased, resulting in defective synaptic pruning in adolescence. Increased mTORC1 also promotes tau aggregation through acetylation and phosphorylation. Furthermore, impaired autophagy prevents the clearance of tau aggregates, ultimately driving synapse loss. Additionally, TS patients exhibit alterations in NKCC1:KCC2 that resemble immature neural circuits. These changes may in part be driven by tau accumulation. Increased NKCC1:KCC2 can alter GABA polarization at inhibitory synapses, but also impairs AMPAR recruitment at excitatory synapses which is necessary for synaptic plasticity associated with learning and memory. Thus, early developmental synaptic alterations likely lead to the accumulation of neurotoxic tau aggregates, impair synaptic plasticity necessary for learning and memory, and alter neuronal excitability, thus driving synapse loss in neurodegenerative disorders. Created with Biorender.com.

In TS mouse models, synapse formation is initially impaired, but synaptic overgrowth is observed later in development (Tavazoie et al., 2005; Phillips and Pozzo-Miller, 2014; Tang et al., 2014; Yasuda et al., 2014). In TS mice, the initial defect in synapse formation corresponds with immature filopodia-like spine precursors (Tavazoie et al., 2005; Yasuda et al., 2014)_._ However, later in development, increased mTORC1 activity impairs macroautophagy of excitatory synapses, resulting in synaptic overgrowth (Tang et al., 2014). Consistent with these temporal differences in synaptic pathology, mTORC1 inhibition fails to rescue the emergence of TS-associated deficits in synapse formation but restores synaptic pruning later in synapse development (Tavazoie et al., 2005; Phillips and Pozzo-Miller, 2014; Tang et al., 2014; Yasuda et al., 2014). Defective macroautophagy may also contribute to the observed increase in excitatory synapses in the temporal lobe of autism patients aged 13–19 years (Tang et al., 2014). Notably, a similar increase is not observed in autism patients of ages 3–9 years, suggesting that TSC-mediated inhibition of mTORC1 is necessary for synaptic pruning to occur at later adolescent stages (Tang et al., 2014). Intriguingly, one might suspect that this synaptic overgrowth might protect TS individuals from age-related FTD (Olney et al., 2017; Liu et al., 2020a). Similar to other neurodegenerative diseases, FTD exhibits synapse loss within the temporal lobe (Clare et al., 2010). However, if the TS-associated mTORC1 hyperactivation prevents synaptic pruning, what are the mechanisms that drive synapse loss and cognitive decline of TS patients later in life? Here, we will explore two potential hypotheses by which developmental TS synaptic pathology later disrupts neural circuits, resulting in their eventual degradation (Figure 1). In the following section, we will first address the mechanisms by which developmental synaptic pathology may contribute to later neurodegeneration and then examine potential therapeutic strategies.

## Potential synaptic mechanisms linking neurodevelopmental and neurodegenerative pathology

### Altered NKCC1:KCC2 in neurodevelopmental and neurodegenerative disorders

As previously mentioned, the ratio of NKCC1 to KCC2 underlies GABA-mediated currents, with developing neural circuits exhibiting higher NKCC1:KCC2 ratios resulting in GABA-induced depolarization and excitation. Intriguingly, TS patients exhibit increased expression of NKCC1 and decreased expression of KCC2, perpetuating GABA-induced excitation as observed in the immature developing brain (Talos et al., 2012; Ruffolo et al., 2016). A human neuronal model of TS recapitulates elevated *SLC12A2* (NKCC1) and decreased *SLC12A5* (KCC2) expression (Costa et al., 2016). The elevated NKCC1:KCC2 ratio may in part be a compensatory mechanism in response to TS-elevated mTORC1 since NKCC1 suppresses mTORC1 activity (Demian et al., 2019). Similar NKCC1:KCC2 alterations are observed in other neurodevelopmental disorders, such as Rett Syndrome (Tang et al., 2016). Neurodegenerative diseases, such as Huntington’s Disease (HD) and possibly Alzheimer’s Disease (AD), exhibit alterations in NKCC1 and KCC2 expression that resemble immature neural circuits (Tang, 2020; Dargaei et al., 2018; Yin et al., 2019/01; Lam et al., 2022; Virtanen et al., 2021). Furthermore, in a human induced pluripotent model of tauopathies, introducing common FTDP-17 mutations (FTD with parkinsonism linked with chromosome 17) reduced *KCC2* expression in differentiated neurons (García-León et al., 2018), further suggesting that reduced KCC2 is a driver of neurodegenerative disease pathology, particularly in tauopathies, such as AD and FTD.

Altered NKCC1:KCC2 expression can impact both synaptic formation and function, potentially contributing to disease-associated hyperexcitability and synapse loss. Acute pharmacological reductions in KCC2 are sufficient to drive hyperexcitability and epileptiform activity through depolarizing GABA currents (Sivakumaran et al., 2015). However, KCC2 also functions independent of GABA at excitatory glutamatergic synapses, where KCC2 is necessary for both dendritic spine maturation and clustering of AMPA receptors (Li et al., 2007; Gauvain et al., 2011). At the dendritic spines of excitatory synapses, reduced KCC2 expression results in immature filopodia-like protrusions and impairs glutamatergic synapse formation (Li et al., 2007), similar to synaptic impairment initially observed in TS (Yasuda et al., 2014). Later in development, TS synapse density increases due to mTORC1-mediated inhibition of autophagy (Tang et al., 2014). However, consistent with KCC2-mediated trafficking of AMPARs, *Tsc1* knockout hippocampal neurons have reduced expression of AMPAR subunits, GluA1/R2 and GluA2/R2 (Bateup et al., 2013). GluA1/2 are specifically added to synapses during periods of plasticity that underlie learning and memory (Shi et al., 2001).

How might early alterations in NKCC1:KCC2 contribute to synaptic alterations in neurodegenerative disorders? Due to the NKCC1:KCC2 regulation of synapse formation and function, GABA-mediated depolarization could contribute to the observed increase in neuronal excitability in neurodegenerative disorders, including FTD (Wishart et al., 2006; Clare et al., 2010; Beagle et al., 2015; Cepeda et al., 2019; Subramanian et al., 2020; Targa Dias Anastacio et al., 2022). Furthermore, GluA1 subunit expression is decreased in neurodegenerative disorders, including AD and FTD, consistent with learning and memory deficits in these disorders (Benussi et al., 2019; Qu et al., 2021). Thus developmental disruptions in synaptic plasticity and activity could predispose individuals to early-onset dementia. Further studies are needed to identify synapse-specific proteomic changes in TS and corresponding synaptic changes in FTD. Identification of synaptic alterations shared between TS and FTD could be used for therapeutic intervention in early development, thus preventing synaptic alterations that likely contribute to later age-related cognitive decline.

Restoring KCC2 expression in TS patients may be complicated as mTORC1 inhibition does not restore the developmental shift in GABA-induced activity; rather, rapamycin treatment decreased KCC2 expression, increasing seizure susceptibility in a juvenile, but not adult, rat model (Huang et al., 2012). Thus, while the rapamycin derivative everolimus reduces patient tumor burden, decreased KCC2 expression could contribute to the persistence of epileptic seizures in ∼50% of treated patients (French et al., 2016; Overwater et al., 2019). Notably, however, these studies were conducted in a chemically-induced seizure model and not in a TS model, where mTORC1 is elevated. Thus, further studies of the effect of mTORC1 inhibition on KCC2 and epilepsy in TS are needed. Nonetheless, the development of KCC2-enhancing drugs may hold promise for restoring GABA-induced inhibition and AMPAR-mediated synaptic plasticity in affected neurodevelopmental and neurodegenerative disorders (Tang et al., 2019; Tang, 2020).

### mTORC1-driven tau post-translational modifications drive pathological accumulation

Since tauopathies, such as AD and FTD, exhibit synapse loss and hyperexcitability, we will next explore how tau pathology may contribute to both neurodevelopmental and neurodegenerative diseases. Pathogenic tau impairs synaptic transmission by binding to the synaptic vesicle protein, synaptogyrin-3, ultimately resulting in the loss of excitatory glutamatergic synapses (Largo-Barrientos et al., 2021). Notably, TS is a tauopathy that exhibits similar pathology to FTD brains, which do not contain amyloid plaques (Liu et al., 2022). Thus, TS is a good model to assess the role of tau aggregation independent of amyloid plaques. Accumulating evidence also suggests that tau aggregation plays an underappreciated role in other neurodevelopmental disorders (Rankovic and Zweckstetter, 2019). Intriguingly, *TSC1* haploinsufficiency increases tau acetylation and accumulation (Alquezar et al., 2021). This is likely through increased mTORC1 activity since mTORC1 directly activates p300 acetyltransferase, which is responsible for tau acetylation (Wan et al., 2017; Alquezar et al., 2021). In addition to acetylation, mTORC1 also regulates tau aggregation through phosphorylation (Caccamo et al., 2013). Since elevated mTORC1 in TS prevents autophagy-mediated clearance of pathogenic tau aggregates (Alquezar et al., 2021), early post-translational tau modifications may progressively lead to the accumulation of tau aggregates and early-onset dementia and neurodegeneration, especially since hyperphosphorylated tau promotes the self-assembly of tau neurofibrillary tangles (Iqbal et al., 2010). Thus, increased tau phosphorylation and acetylation leading to accumulation are likely early biomarkers of neurodevelopmental disorders that contribute to the increased patient susceptibility to neurodegenerative disorders. As previously noted, increased excitability in tauopathies may be driven in part by decreased KCC2 expression as introduction of FTD-associated tau mutations decrease *KCC2* levels in a human neuronal model (García-León et al., 2018). Furthermore, loss of tau reduces network hyperexcitability in AD and seizure models (Holth et al., 2013). However, this rescue is likely driven by multiple factors since tau loss reduces hyperexcitability in the absence of KCC2 function (Holth et al., 2013). Finally, rapamycin reduces tau aggregate burden in mice and human neurons by activating autophagy (Ozcelik et al., 2013; Silva et al., 2020). Additionally, this reduction in tau burden reduces astrogliosis (Ozcelik et al., 2013), which is associated with neuroinflammation (Fleeman and Proctor, 2021), although tau was recently shown to drive synapse loss through pre-synaptic vesicle association independent of neuroinflammation (Largo-Barrientos et al., 2021). Intriguingly, preventing association of tau with the synaptic vesicle protein, synaptogyrin-3, restores synaptic plasticity and could potentially serve as an additional therapeutic strategy for reducing tau neurotoxicity (Largo-Barrientos et al., 2021).

## Concluding remarks

Accumulating evidence suggests that neurodevelopmental disorders, such as TS, place the affected individual at an increased susceptibility to neurodegeneration. In the present piece, we explored potential synaptic mechanisms driving this association (Figure 1). We first examined how persistent alterations in potassium chloride channels may alter neuronal excitability and synaptic plasticity in developing and degenerating networks. Next, we discussed how early mTORC1-driven post-translational modifications to tau promote accumulation and pathological aggregation leading to synapse loss. The accumulated evidence links early impairment in synaptic plasticity with later synapse loss in neurodegeneration, while also highlighting the need for future studies to identify developmental synaptic alterations that drive age-related synapse loss and neurodegeneration. Insights from future studies of TS and FTD will likely have ramifications for other neurodevelopmental and neurodegenerative disorders, where increased mTORC1 signaling is observed (Negraes et al., 2021).

## References

[B1] Alquezar C. Schoch K. M. Geier E. G. Ramos E. M. Scrivo A. Li K. H. (2021b). TSC1 loss increases risk for tauopathy by inducing tau acetylation and preventing tau clearance via chaperone-mediated autophagy. Sci. Adv. 7 (45), eabg3897. 10.1126/sciadv.abg3897 PMC8570595 34739309

[B3] Araya R. Vogels T. P. Yuste R. (2014). Activity-dependent dendritic spine neck changes are correlated with synaptic strength. Proc. Natl. Acad. Sci. U. S. A. 111 (28), E2895–E2904. LP-E2904. 10.1073/pnas.1321869111 24982196 PMC4104910

[B4] Bassetti D. Luhmann H. J. Kirischuk S. (2021). Effects of mutations in TSC genes on neurodevelopment and synaptic transmission. Int. J. Mol. Sci. 22 (14), 7273. 10.3390/ijms22147273 34298906 PMC8305053

[B5] Bateup H. S. Johnson C. A. Denefrio C. L. Saulnier J. L. Kornacker K. Sabatini B. L. (2013). Excitatory/inhibitory synaptic imbalance leads to hippocampal hyperexcitability in mouse models of tuberous sclerosis. Neuron 78 (3), 510–522. 10.1016/j.neuron.2013.03.017 23664616 PMC3690324

[B6] Beagle A. Darwish S. Karageorgiou E. Vossel K. (2015). Seizures and myoclonus in the early stages of frontotemporal dementia. ). Neurology P1.218 84

[B7] Ben-Ari Y. (2006). Basic developmental rules and their implications for epilepsy in the immature brain. Epileptic Disord. 8 (2), 91–102. 16793570

[B8] Ben-Ari Y. (2002). Excitatory actions of gaba during development: The nature of the nurture. Nat. Rev. Neurosci. 3(9):728–739. 12209121 10.1038/nrn920

[B9] Benussi A. Alberici A. Buratti E. Ghidoni R. Gardoni F. Di Luca M. (2019). Toward a glutamate hypothesis of frontotemporal dementia. Front. Neurosci. 13, 304. 10.3389/fnins.2019.00304 30983965 PMC6449454

[B10] Bolton P. F. Clifford M. Tye C. Maclean C. Humphrey A. le Maréchal K. (2015). Intellectual abilities in tuberous sclerosis complex: Risk factors and correlates from the tuberous sclerosis 2000 study. Psychol. Med. 45 (11), 2321–2331. 10.1017/S0033291715000264 25827976

[B11] Caccamo A. Magrì A. Medina D. X. Wisely E. V. López-Aranda M. F. Silva A. J. (2013). mTOR regulates tau phosphorylation and degradation: implications for Alzheimer’s disease and other tauopathies. Aging Cell 12 (3), 370–380. 10.1111/acel.12057 23425014 PMC3655115

[B12] Cepeda C. Oikonomou K. D. Cummings D. Barry J. Yazon V-W. Chen D. T. (2019). Developmental origins of cortical hyperexcitability in Huntington’s disease: Review and new observations. J. Neurosci. Res. 97 (12), 1624–1635. 10.1002/jnr.24503 31353533 PMC6801077

[B13] Cervi F. Saletti V. Turner K. Peron A. Bulgheroni S. Taddei M. (2020). The TAND checklist: A useful screening tool in children with tuberous sclerosis and neurofibromatosis type 1. Orphanet J. Rare Dis. 15 (1), 237. 10.1186/s13023-020-01488-4 32894194 PMC7487732

[B14] Chu-Shore C. J. Major P. Camposano S. Muzykewicz D. Thiele E. A. (2010). The natural history of epilepsy in tuberous sclerosis complex. Epilepsia 51 (7), 1236–1241. 10.1111/j.1528-1167.2009.02474.x 20041940 PMC3065368

[B15] Clare R. King V. G. Wirenfeldt M. Vinters H. V. (2010). Synapse loss in dementias. J. Neurosci. Res. 88 (10), 2083–2090. 10.1002/jnr.22392 20533377 PMC3068914

[B16] Côme E. Heubl M. Schwartz E. J. Poncer J. C. Lévi S. (2019). Reciprocal regulation of KCC2 trafficking and synaptic activity. Front. Cell. Neurosci. 13, 48. 10.3389/fncel.2019.00048 30842727 PMC6391895

[B17] Costa V. Aigner S. Vukcevic M. Sauter E. Behr K. Ebeling M. (2016). mTORC1 inhibition corrects neurodevelopmental and synaptic alterations in a human stem cell model of tuberous sclerosis. Cell Rep. 15 (1), 86–95. 10.1016/j.celrep.2016.02.090 27052171

[B18] Curatolo P. Moavero R. de Vries P. J. Neurological and neuropsychiatric aspects of tuberous sclerosis complex. Lancet. Neurol. 2015 Jul;14(7):733–745. 10.1016/S1474-4422(15)00069-1 26067126

[B19] Dargaei Z. Bang J. Y. Mahadevan V. Khademullah C. S. Bedard S. Parfitt G. M. (2018). Restoring GABAergic inhibition rescues memory deficits in a Huntington's disease mouse model. Proc. Natl. Acad. Sci. U. S. A. 115 (7), E1618-E1626–26. 29382760 10.1073/pnas.1716871115 PMC5816181

[B20] de Vries P. J. Watson P. (2008). Attention deficits in tuberous sclerosis complex (TSC): Rethinking the pathways to the endstate. J. Intellect. Disabil. Res. 52 (Pt 4), 348–357. 10.1111/j.1365-2788.2007.01030.x 18179508

[B21] de Vries P. J. Whittemore V. H. Leclezio L. Byars A. W. Dunn D. Ess K. C. (2015). Tuberous sclerosis associated neuropsychiatric disorders (TAND) and the TAND Checklist. Pediatr. Neurol. 52 (1), 25–35. 10.1016/j.pediatrneurol.2014.10.004 25532776 PMC4427347

[B22] de Vries P. J. Wilde L. de Vries M. C. Moavero R. Pearson D. A. Curatolo P. (2018). A clinical update on tuberous sclerosis complex-associated neuropsychiatric disorders (TAND). Am. J. Med. Genet. C Semin. Med. Genet. 178 (3), 309–320. 10.1002/ajmg.c.31637 30117265 PMC6209538

[B23] Demian W. L. Persaud A. Jiang C. É Coyaud Liu S. Kapus A. (2019). The ion transporter NKCC1 links cell volume to cell mass regulation by suppressing mTORC1. Cell Rep. 27 (6), 1886–1896. e6. 10.1016/j.celrep.2019.04.034 31067471

[B24] Feliciano D. M. Lin T. V. Hartman N. W. Bartley C. M. Kubera C. Hsieh L. (2013/02/26201). A circuitry and biochemical basis for tuberous sclerosis symptoms: From epilepsy to neurocognitive deficits. Int. J. Dev. Neurosci. 31 (7), 667–678. 10.1016/j.ijdevneu.2013.02.008 PMC3830611 23485365

[B25] Feliciano D. M. (2020). The neurodevelopmental pathogenesis of tuberous sclerosis complex (TSC) Front. Neuroanat., 14. 10.3389/fnana.2020.00039 PMC7381175 32765227

[B26] Fleeman R. M. Proctor E. A. (2021).Astrocytic propagation of tau in the context of Alzheimer’s disease Front. Cell. Neurosci., 15. 10.3389/fncel.2021.645233 PMC8010320 33815065

[B27] Franz D. N. Agricola K. Mays M. Tudor C. Care M. M. Holland-Bouley K. (2015). Everolimus for subependymal giant cell astrocytoma: 5-year final analysis. Ann. Neurol. 78 (6), 929–938. 10.1002/ana.24523 26381530 PMC5063160

[B28] Franz D. N. Belousova E. Sparagana S. Bebin E. M. Frost M. Kuperman R. (2013). Efficacy and safety of everolimus for subependymal giant cell astrocytomas associated with tuberous sclerosis complex (EXIST-1): A multicentre, randomised, placebo-controlled phase 3 trial. Lancet (London, Engl. 381 (9861), 125–132. 10.1016/S0140-6736(12)61134-9 23158522

[B29] French J. A. Lawson J. A. Yapici Z. Ikeda H. Polster T. Nabbout R. (2016). Adjunctive everolimus therapy for treatment-resistant focal-onset seizures associated with tuberous sclerosis (EXIST-3): A phase 3, randomised, double-blind, placebo-controlled study. Lancet (London, Engl. 388 (10056), 2153–2163. 10.1016/S0140-6736(16)31419-2 27613521

[B30] García-León J. A. Cabrera-Socorro A. Eggermont K. Swijsen A. Terryn J. Fazal R. (2018). Generation of a human induced pluripotent stem cell–based model for tauopathies combining three microtubule-associated protein TAU mutations which displays several phenotypes linked to neurodegeneration. Alzheimer’s Dement. 14(10):1261–1280. 30036493 10.1016/j.jalz.2018.05.007

[B31] Gauvain G. Chamma I. Chevy Q. Cabezas C. Irinopoulou T. Bodrug N. (2011). The neuronal K-Cl cotransporter KCC2 influences postsynaptic AMPA receptor content and lateral diffusion in dendritic spines. Proc. Natl. Acad. Sci. U. S. A. 108 (37), 15474–15479. 10.1073/pnas.1107893108 21878564 PMC3174661

[B32] Giannikou K. Lasseter K. D. Grevelink J. M. Tyburczy M. E. Dies K. A. Zhu Z. (2019). Low-level mosaicism in tuberous sclerosis complex: Prevalence, clinical features, and risk of disease transmission. Genet. Med. 21 (11), 2639–2643. 10.1038/s41436-019-0562-6 31160751

[B33] Hahn C-G. Banerjee A. Macdonald M. L. Cho D-S. Kamins J. Nie Z. (2009/04/16200). The post-synaptic density of human postmortem brain tissues: An experimental study paradigm for neuropsychiatric illnesses. PLoS One 4 (4), e5251. 10.1371/journal.pone.0005251 PMC2666803 19370153

[B34] Holth J. K. Bomben V. C. Reed J. G. Inoue T. Younkin L. Younkin S. G. (2013). Tau loss attenuates neuronal network hyperexcitability in mouse and Drosophila genetic models of epilepsy. J. Neurosci. 33 (4), 1651–1659. 10.1523/JNEUROSCI.3191-12.2013 23345237 PMC3711605

[B35] Huang X. McMahon J. Yang J. Shin D. Huang Y. (2012). Rapamycin down-regulates KCC2 expression and increases seizure susceptibility to convulsants in immature rats, Neuroscience 219, 33–47. 22613737 10.1016/j.neuroscience.2012.05.003 PMC3402618

[B36] Iqbal K. Liu F. Gong C-X. Grundke-Iqbal I. (2010). Tau in Alzheimer disease and related tauopathies. Curr. Alzheimer Res. 7 (8), 656–664. 10.2174/156720510793611592 20678074 PMC3090074

[B37] Kingswood J. C. d’Augères G. B. Belousova E. Ferreira J. C. Carter T. Castellana R. (2017). TuberOus SClerosis registry to increase disease Awareness (TOSCA) – baseline data on 2093 patients. Orphanet J. Rare Dis. 12 (1), 2. 10.1186/s13023-016-0553-5 28057044 PMC5217262

[B38] Krueger D. A. Sadhwani A. Byars A. W. de Vries P. J. Franz D. N. Whittemore V. H. (2017). Everolimus for treatment of tuberous sclerosis complex-associated neuropsychiatric disorders. Ann. Clin. Transl. Neurol. 4 (12), 877–887. 10.1002/acn3.494 29296616 PMC5740257

[B39] Krueger D. A. Wilfong A. A. Holland-Bouley K. Anderson A. E. Agricola K. Tudor C. (2013). Everolimus treatment of refractory epilepsy in tuberous sclerosis complex. Ann. Neurol. 74 (5), 679–687. 10.1002/ana.23960 23798472

[B40] Lam P. Vinnakota C. Guzmán B. C-F. Newland J. Peppercorn K. Tate W. P. (2022). Beta-amyloid (Aβ(1-42)) increases the expression of NKCC1 in the mouse Hippocampus. Molecules 27 (8), 2440. 10.3390/molecules27082440 35458638 PMC9027496

[B41] Laplante M. Sabatini D. M. mTOR signaling in growth control and disease. Cell. 2012 Apr;149(2):274–293. 10.1016/j.cell.2012.03.017 22500797 PMC3331679

[B42] Largo-Barrientos P. Apóstolo N. Creemers E. Callaerts-Vegh Z. Swerts J. Davies C. (2021). Lowering Synaptogyrin-3 expression rescues Tau-induced memory defects and synaptic loss in the presence of microglial activation. Neuron 109 (5), 767–777.e5. e5. 10.1016/j.neuron.2020.12.016 33472038 PMC7927913

[B43] Lechuga L. Franz D. N. (2019). Everolimus as adjunctive therapy for tuberous sclerosis complex-associated partial-onset seizures. Expert Rev. Neurother. 19 (10), 913–925. 10.1080/14737175.2019.1635457 31335226

[B44] Li H. Khirug S. Cai C. Ludwig A. Blaesse P. Kolikova J. (2007). KCC2 interacts with the dendritic cytoskeleton to promote spine development. Neuron 56 (6), 1019–1033. 10.1016/j.neuron.2007.10.039 18093524

[B45] Lipton J. O. Sahin M. (2014). The neurology of mTOR. Neuron 84 (2), 275–291. 10.1016/j.neuron.2014.09.034 25374355 PMC4223653

[B46] Liu A. J. Lusk J. B. Ervin J. Burke J. O’Brien R. Wang S-H. J. (2022). Tuberous sclerosis complex is a novel, amyloid-independent tauopathy associated with elevated phosphorylated 3R/4R tau aggregation. Acta Neuropathol. Commun. 10 (1), 27. 10.1186/s40478-022-01330-x 35241183 PMC8896101

[B48] Liu A. J. Staffaroni A. M. Rojas-Martinez J. C. Olney N. T. Alquezar-Burillo C. Ljubenkov P. A. (2020a). Association of cognitive and behavioral features between adults with tuberous sclerosis and frontotemporal dementia. JAMA Neurol. 77 (3), 358–366. 10.1001/jamaneurol.2019.4284 31860018 PMC6990672

[B49] Liu R. Wang J. Liang S. Zhang G. Yang X. (2020b). Role of NKCC1 and KCC2 in epilepsy: From expression to function [internet] Front. Neurology, 10. 10.3389/fneur.2019.01407 PMC6978738 32010056

[B50] Lynch G. Rex C. S. Gall C. M. (2007). LTP consolidation: Substrates, explanatory power, and functional significance. Neuropharmacol. 52(1):12–23. 10.1016/j.neuropharm.2006.07.027 16949110

[B51] Murmu R. P. Li W. Szepesi Z. Li J. Y. Altered sensory experience exacerbates stable dendritic spine and synapse loss in a mouse model of huntington's disease. J. Neurosci. . 2015 35(1):287 LP – 298. 25568121 10.1523/JNEUROSCI.0244-14.2015 PMC6605245

[B52] Negraes P. D. Trujillo C. A. Yu N-K. Wu W. Yao H. Liang N. (2021). Altered network and rescue of human neurons derived from individuals with early-onset genetic epilepsy. Mol. Psychiatry 26 (11), 7047–7068. 10.1038/s41380-021-01104-2 33888873 PMC8531162

[B53] Newell-Litwa K. A. K. A. Badoual M. Asmussen H. Patel H. Whitmore L. Horwitz A. R. A. R. (2015). ROCK1 and 2 differentially regulate actomyosin organization to drive cell and synaptic polarity. J. Cell Biol. 210 (2), 225–242. 10.1083/jcb.201504046 26169356 PMC4508895

[B54] Olney N. T. Alquezar C. Ramos E. M. Nana A. L. Fong J. C. Karydas A. M. (2017). Linking tuberous sclerosis complex, excessive mTOR signaling, and age-related neurodegeneration: A new association between TSC1 mutation and frontotemporal dementia. Acta Neuropathol. 134, 813–816. 10.1007/s00401-017-1764-0 28828560 PMC5645431

[B55] Overwater I. E. Rietman A. B. van Eeghen A. M. de Wit M. C. Y. (2019). Everolimus for the treatment of refractory seizures associated with tuberous sclerosis complex (TSC): Current perspectives. Ther. Clin. Risk Manag. 15, 951–955. 10.2147/TCRM.S145630 31440057 PMC6666377

[B56] Ozcelik S. Fraser G. Castets P. Schaeffer V. Skachokova Z. Breu K. (2013). Rapamycin attenuates the progression of tau pathology in P301S tau transgenic mice. PLoS One 8 (5), e62459. 10.1371/journal.pone.0062459 PMC3646815 23667480

[B57] Penzes P. Cahill M. E. Jones K. A. VanLeeuwen J-E. Woolfrey K. M. (2011). Dendritic spine pathology in neuropsychiatric disorders. Nat. Neurosci. 14 (3), 285–293. 10.1038/nn.2741 21346746 PMC3530413

[B59] Phillips M. Pozzo-Miller L. (2014). Neuroscience letters,Dendritic spine dysgenesis autism Relat. Disord. 601. Elsevier Ireland Ltd, 30–40. 10.1016/j.neulet.2015.01.011 PMC4496332 25578949

[B60] Qu W. Yuan B. Liu J. Liu Q. Zhang X. Cui R. (2021). Emerging role of AMPA receptor subunit GluA1 in synaptic plasticity: Implications for Alzheimer’s disease. Cell Prolif. 54 (1), e12959. 10.1111/cpr.12959 PMC7791177 33188547

[B61] Rankovic M. Zweckstetter M. (2019). Upregulated levels and pathological aggregation of abnormally phosphorylated Tau-protein in children with neurodevelopmental disorders. Neurosci. Biobehav. Rev. 98, 1–9. 10.1016/j.neubiorev.2018.12.014 30550860

[B62] Ruffolo G. Iyer A. Cifelli P. Roseti C. Mühlebner A. van Scheppingen J. (2016). Functional aspects of early brain development are preserved in tuberous sclerosis complex (TSC) epileptogenic lesions, Neurobiol. Dis. 95, 93–101. 27425893 10.1016/j.nbd.2016.07.014

[B63] Sedmak G. Jovanov-Milošević N. Puskarjov M. Ulamec M. Krušlin B. Kaila K. (2016). Developmental expression patterns of KCC2 and functionally associated molecules in the human brain. Cereb. Cortex 26 (12), 4574–4589. [Internet]Available from:. 10.1093/cercor/bhv218 26428952

[B64] Sernagor E. Chabrol F. Bony G. Cancedda L. (2010).GABAergic control of neurite outgrowth and remodeling during development and adult neurogenesis: General rules and differences in diverse systems [internet] Front. Cell. Neurosci., 4. 10.3389/fncel.2010.00011 PMC2859806 20428495

[B65] Shepherd C. W. Stephenson J. B. P. (1992). Seizures and intellectual disability associated with tuberous sclerosis complex in the west of scotland. Dev. Med. Child. Neurol. 34 (9), 766–774. 10.1111/j.1469-8749.1992.tb11515.x 1526347

[B66] Shi S-H. Hayashi Y. Esteban J. A. Malinow R. (2001). Subunit-specific rules governing AMPA receptor trafficking to synapses in hippocampal pyramidal neurons. Cell 105 (3), 331–343. Cell 10.1016/S0092-8674(01)00321-X 11348590

[B67] Silva M. C. Nandi G. A. Tentarelli S. Gurrell I. K. Jamier T. Lucente D. (2020). Prolonged tau clearance and stress vulnerability rescue by pharmacological activation of autophagy in tauopathy neurons. Nat. Commun. 11 (1), 3258. 10.1038/s41467-020-16984-1 32591533 PMC7320012

[B68] Sivakumaran S. Cardarelli R. A. Maguire J. Kelley M. R. Silayeva L. Morrow D. H. (2015). Selective inhibition of KCC2 leads to hyperexcitability and epileptiform discharges in hippocampal slices and *in vivo* . J. Neurosci. 35(21):8291–8296. 26019342 10.1523/JNEUROSCI.5205-14.2015 PMC4444547

[B69] Subramanian J. Savage J. C. Tremblay M-È. (2020). Synaptic loss in alzheimer's disease: Mechanistic insights provided by two-photon *in vivo* imaging of transgenic mouse models. Front. Cell. Neurosci. 14, 445. 10.3389/fncel.2020.592607 PMC7780885 33408613

[B70] Talos D. M. Sun H. Kosaras B. Joseph A. Folkerth R. D. Poduri A. (2012). Altered inhibition in tuberous sclerosis and type IIb cortical dysplasia. Ann. Neurol. 71 (4), 539–551. 10.1002/ana.22696 22447678 PMC3334406

[B71] Tang B. L. (2020). The expanding therapeutic potential of neuronal KCC2. Cells 9 (1), E240. 10.3390/cells9010240 PMC7016893 31963584

[B72] Tang G. Gudsnuk K. Kuo S-H. Cotrina M. L. Rosoklija G. Sosunov A. (2014). Loss of mTOR-dependent macroautophagy causes autistic-like synaptic pruning deficits. Neuron [Internet] .[cited 2017 May 5];83(5):1131–1143. Available from:25155956 10.1016/j.neuron.2014.07.040 PMC4159743

[B73] Tang X. Drotar J. Li K. Clairmont C. D. Brumm A. S. Sullins A. J. (2019). Pharmacological enhancement of *KCC2* gene expression exerts therapeutic effects on human Rett syndrome neurons and *Mecp2* mutant mice. Sci. Transl. Med. 11 (503), eaau0164. eaau0164. 31366578 10.1126/scitranslmed.aau0164 PMC8140401

[B74] Tang X. Kim J. Zhou L. Wengert E. Zhang L. Wu Z. (2016). KCC2 rescues functional deficits in human neurons derived from patients with Rett syndrome. Proc. Natl. Acad. Sci. 113(3):751–756. 26733678 10.1073/pnas.1524013113 PMC4725523

[B75] Targa Dias Anastacio H. Matosin N. Ooi L. (2022). Neuronal hyperexcitability in Alzheimer’s disease: What are the drivers behind this aberrant phenotype? Transl. Psychiatry 12 (1), 257. 10.1038/s41398-022-02024-7 35732622 PMC9217953

[B76] Tau G. Z. Peterson B. S. (2010). Normal development of brain circuits. Neuropsychopharmacol. 35(1):147–168. 10.1038/npp.2009.115 PMC3055433 19794405

[B77] Tavazoie S. F. Alvarez V. A. Ridenour D. A. Kwiatkowski D. J. Sabatini B. L. (2005). Regulation of neuronal morphology and function by the tumor suppressors Tsc1 and Tsc2. Nat. Neurosci. 8 (12), 1727–1734. 10.1038/nn1566 16286931

[B78] Tønnesen J. Nägerl U. V. (2016). Dendritic spines as tunable regulators of synaptic signals. Front. Psychiatry 7, 101. [Internet]Available from:. 10.3389/fpsyt.2016.00101 27340393 PMC4899469

[B79] Tran L. H. Zupanc M. L. (2015). Long-term everolimus treatment in individuals with tuberous sclerosis complex: A review of the current literature. Pediatr. Neurol. 53 (1), 23–30. Pediatr Neurol [Internet]Available from:. 10.1016/j.pediatrneurol.2014.10.024 26092412

[B80] Verhoef S. Bakker L. Tempelaars A. M. Hesseling-Janssen A. L. Mazurczak T. Jozwiak S. (1999). High rate of mosaicism in tuberous sclerosis complex. Am. J. Hum. Genet. 64 (6), 1632–1637. 10.1086/302412 10330349 PMC1377905

[B81] Virtanen M. A. Uvarov P. Mavrovic M. Poncer J. C. Kaila K. (2021). The multifaceted roles of KCC2 in cortical development. Trends Neurosci. 44 (5), 378–392. Available from:. 10.1016/j.tins.2021.01.004 33640193

[B82] Vivanti G. Tao S. Lyall K. Robins D. L. Shea L. L. (2021). The prevalence and incidence of early-onset dementia among adults with autism spectrum disorder. Autism Res. 14 (10), 2189–2199. 10.1002/aur.2590 34378867 PMC8487995

[B83] Wan W. You Z. Xu Y. Zhou L. Guan Z. Peng C. (2017). mTORC1 phosphorylates acetyltransferase p300 to regulate autophagy and lipogenesis. Mol. Cell 68 (2), 323–335. e6. 10.1016/j.molcel.2017.09.020 29033323

[B84] Wilson E. S. Newell-Litwa K. (2018). “Stem cell models of human synapse development and degeneration,” Mol. Biol. Cell [Internet. Editor M Bronner, 29, 2913–2921. Available from: .24 30475098 10.1091/mbc.E18-04-0222 PMC6329912

[B85] Wishart T. M. Parson S. H. Gillingwater T. H. Synaptic vulnerability in neurodegenerative disease. J. Neuropathol. Exp. Neurol. 65, 733–739. 2006 10.1097/01.jnen.0000228202.35163.c4 16896307

[B86] Yasuda S. Sugiura H. Katsurabayashi S. Shimada T. Tanaka H. Takasaki K. (2014). Activation of Rheb, but not of mTORC1, impairs spine synapse morphogenesis in tuberous sclerosis complex. Sci. Rep. 4, 5155. Sci Rep [Internet]Available from:. 10.1038/srep05155 24889507 PMC4042127

[B87] Yin C. Ackermann S. Ma Z. Mohanta S. K. Zhang C. Li Y. (2019/01/28201). ApoE attenuates unresolvable inflammation by complex formation with activated C1q. Nat. Med. 25 (3), 496–506. [Internet]Available from:. 10.1038/s41591-018-0336-8 PMC6420126 30692699

